# 4306 Periodontal disease and the oral microbiome in antiretroviral-treated patients with HIV

**DOI:** 10.1017/cts.2020.309

**Published:** 2020-07-29

**Authors:** Medini K Annavajhala, Jayesh G. Shah, Jessica Weidler, Karolina Kister, Ryan T. Demmer, Sunil Wadhwa, Michael T. Yin, Anne-Catrin Uhlemann

**Affiliations:** 1 Columbia University, Irving Institute for Clinical; 2 Columbia University Irving Medical Center; 3 University of Minnesota

## Abstract

OBJECTIVES/GOALS: People living with HIV, despite antiretroviral therapy (ART), have increased burden of inflammatory and aging-related comorbidities such as periodontitis. Oral microbiota have been linked to periodontitis, but not in the context of HIV. We aim to compare relationships between the oral microbiome and periodontal disease in HIV+ vs healthy controls. METHODS/STUDY POPULATION: In an ongoing cohort study we have been recruiting pre- and post-menopausal women with HIV+ on ART for ≥6 months and HIV- controls matched by menopausal status (target n = 30 per arm; currently HIV+: n = 30 post- and 9 pre-M; HIV-: n = 15 post- and 6 pre-M). Patients age <18 or on antibiotics within 3 mos., except prophylaxis, are excluded. Patients provide saliva, then subgingival plaque collection during a dental examination through scaling from six index teeth. Standard CDC/AAP classifications of periodontitis are used. We will perform 16S rRNA and ITS sequencing to profile bacterial and fungal communities in saliva and plaque. Linear mixed effect regression and differential abundance analyses will be used to identify microbial and mycobial oral signatures of periodontal disease severity in HIV+ and HIV- populations. RESULTS/ANTICIPATED RESULTS: We found a markedly high prevalence of severe periodontal disease in HIV+ women despite ART (59%, compared to 11% in HIV- controls). In post-menopausal women with HIV, saliva bacterial α- and β-diversity in the saliva differed significantly with periodontal disease severity. Fungal α-diversity was also significantly lower in plaque from teeth with severe loss of tissue attachment (CAL ≥4 mm). We identified bacterial and fungal taxa significantly enriched in post-menopausal HIV+ women with severe compared to no or mild periodontitis. We hypothesize, similarly, associations between the oral microbiome and periodontitis in HIV- controls. However, we expect overall diversity metrics to be significantly altered in HIV+ compared to HIV- patients, indicating long-term dysbiosis despite treatment with ART. DISCUSSION/SIGNIFICANCE OF IMPACT: Contrasting associations between the oral microbiome and periodontal disease with respect to HIV will provide evidence for the role of microbiota in accelerated aging phenotype caused by HIV. Our results would also provide rationale for interventions targeting co-morbidities in people living with HIV to account for both inflammation and dysbiosis.

